# Titrating droxidopa to maximize symptomatic benefit in a patient with Parkinson disease and neurogenic orthostatic hypotension

**DOI:** 10.1007/s10286-017-0430-x

**Published:** 2017-07-11

**Authors:** Fiona Gupta, Beverly Karabin, Ali Mehdirad

**Affiliations:** 1 Movement Disorders Center, Hackensack University Medical Center, Hackensack, NJ USA; 2 Syncope and Autonomic Disorders Center, University of Toledo, Toledo, OH USA; 3 Cardiac Electrophysiology Section, Division of Cardiology, Department of Internal Medicine, Saint Louis University, St. Louis, MO USA

**Keywords:** Droxidopa, Neurogenic orthostatic hypotension

## Challenge questions

What is the optimal method to titrate droxidopa to achieve the maximal symptomatic benefit? How should clinicians monitor for supine hypertension?

## Case presentation

Mr. E is an 80-year-old male with well-controlled Parkinson disease (PD). He was able to walk 1 mile daily until he developed occasional episodes of dizziness and syncope when standing. His nurse practitioner detected a significant fall in blood pressure (BP) when standing without an adequate heart rate (HR) increase [seated BP of 120/70 mmHg with a HR of 66 beats per minute (bpm); standing BP of 89/60 mmHg with a HR of 68 bpm]. He was therefore diagnosed with symptomatic neurogenic orthostatic hypotension (nOH). He was prescribed midodrine [2], fludrocortisone, and pyridostigmine. However, he reported little or no benefit with any of them. He reported that he continues to suffer from symptoms of nOH, including fatigue, weakness, lightheadedness, and near syncope upon standing. He has become wheelchair-ridden many days, and his wife is concerned that she is unable to care for him because of his dizziness causing falls. His wife also reported that her husband has been reluctant to engage in previous activities such as attending worship services, exercise, and bingo games, instead preferring to sit and watch television. Orthostatic vital signs measured in the office revealed marked nOH with supine BP of 130/70 mmHg and 3-min standing BP of 90/60 mmHg. Supine HR was 68 bpm, and standing HR was 64 bpm. It was determined that the patient is a good candidate for treatment with droxidopa [1].

The patient and his wife were educated on home BP monitoring. They were instructed to check his BPs in the supine position (at least 15 min after lying down at bedtime or prior to arising from bed in the morning) and after 3 min of standing (with assistance as necessary) after arising from bed in the morning. They were instructed that supine BP measurements are to be performed with a 30° elevation of the head of the bed. If significant supine hypertension (sHTN) (systolic BP ≥180 mmHg) is recorded, they were advised to measure and record the BP three times in 1 h to see if this was sustained.

The patient was initiated on 100 mg droxidopa on a modified three times daily (TID) schedule, with first dose in the morning, second dose at midday, and the last dose at least 3–4 h prior to bedtime. If significant sHTN (systolic BP ≥180 mmHg) is not recorded, the patient was instructed to up-titrate droxidopa by 100 mg on the modified TID schedule (morning, lunchtime, and 3–4 h before bedtime) every 24–48 h based on symptomatic improvement. They were directed to measure Mr. E’s BPs in the supine position at least twice daily (morning and before bedtime) while in the titration phase and measure his BP if he was feeling symptomatic. The patient was otherwise instructed to continue titration (up to a maximum dose of 600 mg on the modified TID schedule) until achieving symptomatic improvement in activities, stand time, and overall improvement in weakness, lightheadedness, and/or near syncope.

## Expert commentary (Dr. Karabin)

There are a number of patient characteristics influencing the titration rate; these may include sHTN, cardiovascular disease, cerebrovascular disease, advanced neurological disease (e.g., dementia, PD), and elderly with limited home support. In all of these cases, titration may be slower (e.g., every 72–96 h).

## Expert commentary (Dr. Gupta)

Education is key to making the proper decision for titration. If a patient wants to titrate more slowly, I would advise against this, especially in a very symptomatic patient with nOH; this is because slowing the titration prolongs the time until the beneficial impact that droxidopa can play on symptoms of nOH. However, every patient should be managed based on their comorbidities, ability to tolerate treatment, and age. Thus, some patients with more comorbidities may require a slower titration.

## Case continuation

The patient reported that after receiving 400 mg droxidopa TID, his symptoms were much improved with very few episodes of dizziness. The patient continued this dosage schedule until the next follow-up visit, after approximately 4 weeks of starting droxidopa.

At the next follow-up visit 3 months later (supine BP of 134/74 mmHg with a HR of 60 bpm; 3-min standing BP of 115/68 mmHg with a HR of 67 bpm), the wife reported that her husband had experienced a few episodes of dizziness and had fallen once. At the visit, the patient was instructed to again up-titrate the dose of droxidopa every 24–48 h up to a maximum dose of 600 mg on the modified TID schedule, as mentioned above. In addition, they were instructed to monitor his BPs, as before, during the titration phase.

After achieving the maximum dose of 600 mg TID, the patient and his wife reported that his symptoms were much improved. He has not had any episodes of falling during the past 3 months and has only had a couple of episodes of dizziness.
